# Economic growth for ecological conversions: South Korean case

**DOI:** 10.1186/s12302-018-0149-x

**Published:** 2018-06-18

**Authors:** Hye Kyung Lee, Hwan Yong Kim

**Affiliations:** 1 Super-Tall Building Global R&D Center, Dankook University, 152, Jukjeon-ro, Yongin-si, South Korea; 2 Division of Architecture & Urban Design College of Urban Sciences, Incheon National University, 119 Academy-Ro, Yeonsu-Gu, Incheon, 406-772 South Korea

**Keywords:** Ecological valuation, Urban sprawl, Economic growth, Environmental justice, Land cover, Geographic information systems

## Abstract

**Background:**

Sprawl has been named as one of the critical reasons for the latest social problems in many parts of the world. This is particularly true for developing countries, as their national status largely depends on economic stability and interacts with the rise and decline of major cities. This study focuses on a detailed notion on environmental impact of physical expansion and answers how to specifically estimate the ecological impact of sprawl using the GIS and ecological valuation method. Especially, South Korean cities are examined to identify how development-oriented growth would affect natural stock and the ecology as a whole.

**Results:**

By implementing land cover datasets and an estimation method, value transfer, the authors examine the economic losses of Korean ecological stock between 1980 and 2000. Since 1980, the society has gained a significant amount of growth in its national economics. Specifically, GDP has increased from about $40 billion to $640 billion. However, due to its rapid growth, the entire natural stock has lost about 5% of its total features, using the median economic values. If calculated with the maximum values, it is about a 7% decrease. The results indicate that $2076/person for environmental opportunity costs is estimated as a consequence of rapid urbanization.

**Conclusions:**

If we had estimated the ecological consumptions of rapid growth from the beginning and considered $2076/person for environmental opportunity costs, then the development patterns and other associated urban planning agendas would have shifted accordingly to increase the overall sustainability. Like most developing cities in the world, major cities in South Korea and the central government concentrated its main strategy on economic growth. Doing so stimulated national economy and made it possible to level up the quality of life. If this quality of life needs to be sustained for a long term, then we should focus on our usage of ecological features, as their characteristics are completely different from man-made resources.

## Background

Sprawl has been named as one of the critical reasons for the latest social and urban problems in many part of the world. Accordingly, it is a widely studied subject across diverse disciplines [12, 18, 20, 24, 41, 52]. This is particularly true in developing countries, as their national status largely depends on economic stability and strongly interacts with the rise and decline of major cities. As a consequence, a large number of studies have elaborated the effects of urban sprawl, which can be summarized into six aspects: (1) effects of sprawl on public health [3, 14, 19, 23, 35, 44]; (2) on real estate market [4, 6, 42, 43]; (3) on transportation [15–17, 39, 40, 45]; (4) on land use [13, 22, 27, 30, 39]; (5) on the environment [7, 26, 29, 46]; and (6) on urban population [21].

Of those different perspectives, this study focuses on a more detailed notion of the environmental impact of physical expansion. Because of its unpredictable nature, environmental impact of urban sprawl is often studied using limited externalities such as air pollution, congestion, safety, or noise for the aggregate study areas. The shortcoming of such an approach is that the physical expansion’s specific impact on the ecological features for the selected geographic boundary is generally left behind. To this extent, this study answers how to specifically estimate the ecological impact of sprawl using the geographic information systems (GIS) and ecological valuation method. Especially, South Korean cities are examined to identify how development-oriented direction that is still prevalent in most developing countries would affect natural stock and the ecology as a whole. By implementing different years of land cover datasets and an estimation method, value transfer, the authors examine the economic losses of Korean ecological stock since the 1980s through the 2000s. Doing so provides a brief outline about what are the environmental consequences and responsibilities of becoming one of the fastest growing countries in the world.

## Methods

### Urban sprawl and environmental externalities: the South Korean case

Like many other developing cities in the world, major cities in South Korea have experienced a massive expansion over the past decades. Many reasons can be accounted for this, but during the 1990s it was obvious that the Korean government mainly focused its national strategy on economic growth [32, 36]. Since 1950 when the Korean War ended, it was undoubtedly clear that the socioeconomic status of South Korea was in its deepest downturn. As a response, government officials and associated experts dedicated themselves to improving economic status and concentrating on quantitative growth [37]. This type of approach works mainly for the major cities where the necessary socioeconomic infrastructure is maintained. For that reason, national growth strategy was heavily geared toward large cities across the country.

Specifically, the South Korean government has implemented two major urban developments. Starting in the mid-1990s, the government has constructed new cities in the vicinity of the capital, Seoul. Named as the first generation of the new city model, about six new cities were constructed to accommodate approximately a 1,740,000 population, accounting for about 623,000 households [34]. This development pattern is slightly different from the typical sprawl patterns that took place in the Western Hemisphere, as the density of the new cities was completely different. Because of job–housing imbalance, however, the negative consequences of urban sprawl were found in a similar format. A diverse range of impacts of sprawl such as traffic congestion, travel time increase, carbon emissions, expanded social costs and so forth [5] were detected. Although the first-generation cities were suggested to invigorate national growth and support the massive population expansion, socioeconomic problems were generated in the same dimension as the majority of the world.

To ease the negative consequences of the first-generation cities, the government decided to try a new type of city development and named it the second-generation city model [34]. Unlike its precursors, the second model used a number of different approaches to mingle jobs and housing together in the same political boundary. Strategies such as tax exemption, financial assistance, and property long-term lease were identified to encourage housing–job balance. However, the problem stemmed from the point where housing developments and residential movements happened relatively easier and faster, whereas job relocation was a much slower process [5]. Although the degree of the impact has changed, the South Korean society is again experiencing similar negative externalities of urban sprawl.

Entering the twenty-first century meant a new era for Korean society to pay less attention to its physical growth, but accentuate more on the quality side of its expansion [31]. A number of international indices, such as the Organization for Economic Cooperation and Development (OECD) Better Life Index, have experienced a rapid improvement based on aggressive economic growth [38]. Nonetheless, it was still unclear whether the nation’s development direction fell well with the overall quality of citizens’ life. In addition, more attention has been paid to the environment since the beginning of the twenty-first century, and the degree of our development speed and the amount of physical conversion have been reviewed by many professionals to gauge the overall sustainability of the society [36, 37]. In this context, it would be helpful to analyze how much environmental consumption took place during the growth and understand what urban expansion means in terms of ecological opportunity costs. By doing so, the degree of economic responsibility of converting natural environment into physical expansion could be elaborated.

### Ecological value transfer

To capture the details of the associated environmental opportunity costs, this study models land cover difference of South Korea in 1980, 1990, and 2000. The amount of pervious lands converted into impervious surfaces is calculated for 1980, 1990, and 2000. After that, each land cover type is estimated into monetary values to provide the overall costs of any associated ecological losses.

Ecological cost is estimated using an approach known as value transfer. Value transfer is one particular methodology in the discipline of ecosystem valuation. Although location-specific or micro-level valuation studies are in demand, they generally require more intensive datasets and more precise measurements than studies at an aggregate level [33, 50]. Thus, data availability often becomes an issue. To overcome the limitations and keep the focus on project-specific value measurements, researchers in the ecosystem science suggest a secondary analysis method: value transfer [28, 33, 47, 50]. This transfer method involves obtaining an estimate for the economic value of non-market goods or services through the analysis of a single study or group of studies that have been previously carried out to value similar goods or services. When conducting a primary research work where accurate data collection is not strongly feasible, value transfer represents a meaningful “second-best” strategy and starting point for the evaluation of environmental features [10, 25, 28, 50, 51].

Estimating ecological cost using value transfer involves a number of steps. First, land cover types within the study area should be identified, and there are six types in this study. After that, relevant literature works are collected and summarized to identify transferrable values for each land cover. There are various studies and databases delineating the economic values of land covers such as Environmental Valuation Reference Inventory (EVRI), the Ecosystem Services Partnership, and Natural Capital Project. These types of databases with the study results have increased in the past years [1, 2, 9–11, 49, 50], and the validity of assessing the economic costs of each land cover has become more stable.

For this study, 88 cases and 51 relevant articles were examined. Table 1 summarizes six land cover types in the entire South Korea and their corresponding monetizable elements. 88 studies have been closely examined to identify the economic values of each land cover with the corresponding services, and the services provided by the six land covers can be summarized into: (1) climate regulation; (2) water supply; (3) recreation; (4) habitat; (5) pollination; and (6) soil formation. As can be seen, many studies have focused on the economic values of forests and wetlands, resulting in 54 studies, and many have articulated the ecological benefits of water supply and esthetic values, resulting in 38 cases.

**Table 1:** Value transfer studies summary

Land cover	Economic values							Total
	Overall estimate	Climate regulation	Water supply and regulation	Recreation and esthetic	Habitat refuge	Pollination	Soil formation and control	
Open water	2	–	2	2	1	–	–	7
Forest	3	6	3	4	9	2	–	27
Herbaceous	1	2	3	1	1	1	2	11
Pasture	2	–	–	1	–	–	1	4
Crop	3	3	1	3	1	–	1	12
Wetland	3	1	10	8	5	–	–	27
Total	14	12	19	19	17	3	4	88

Based on these records, Table 2 summarizes the median, mean, maximum, and minimum values of each land cover. Some ecological features show significant cost variances across their minimum and maximum values. For example, if one acre of wetland is converted into an impervious surface, then the expected minimum economic loss is about $0.39/year, whereas the maximum can go as high as $144,636/year. This is a substantial difference and cannot be considered a reliable measure. The minimum values are estimated in a very conservative manner and, for that reason, most of the minimum values are not practical estimates. For example, it is considered that one tree could make about 8300 sheets of paper, which is approximately eight reams [8], and one ream of paper is sold in a range of $3–$8 in major retail sources. However, the minimum economic cost of converting one acre of forest land is $0.18 according to previous studies, suggesting that using the median values would be more reliable.

**Table 2:** Economic values ($/acre/year) for each land cover type

Land cover types	Median	Mean	Minimum	Maximum
Open water	$876.72	$3,375.66	$1.76	$21,817.25
Forest	$245.84	$1,102.47	$0.18	$10,738.07
Herbaceous	$15.84	$76.22	$1.90	$355.73
Pasture	$906.34	$3,214.40	$0.03	$11,044.90
Crop	$22.4	$909.22	$2.59	$6,608.18
Wetland	$1,437.89	$8,420.64	$0.39	$144,635.79

All of the 88 studies have articulated different economic values of each land cover and, thus, their monetizable values are heterogeneous in nature. In this context, it would be appropriate to implement more scrutiny in valuing each land cover. For example, it would be helpful to use the 75‰ values for each land cover, if the number of studies is abundant. However, some land covers, such as the wetlands, have more than 25 studies, whereas some covers, such as open waters, have only 7 study results of their economic values. Therefore, it is hard to specifically identify the majority of values for each cover and, for that reason, the median values would be more logical to use in terms of land cover monetization.

Another piece of information to estimate the opportunity costs of ecological consumptions is acreage. As described in Table 2, the value estimates are based on acreage per year. It means each land cover’s acreage information should be assessed prior to cost estimation. Using GIS land cover dataset, it is possible to pinpoint how much natural land covers are converted to impervious surfaces. The Ministry of Environment in South Korea provides historic land cover datasets, and in this case 30M × 30M land cover is used to calculate the land cover areas. Economic values using the medians are set into per acre information and each land cover’s acreage information could be converted with the number of 30M pixels inside the land cover’s boundary. Once the necessary information is set, GIS can easily calculate the total monetizable values of each land cover by multiplying per acre median values of each land cover with the total acre of each land cover.

## Results and discussion

### Land cover changes in Korea between 1980 and 2000

**Fig. 1:**
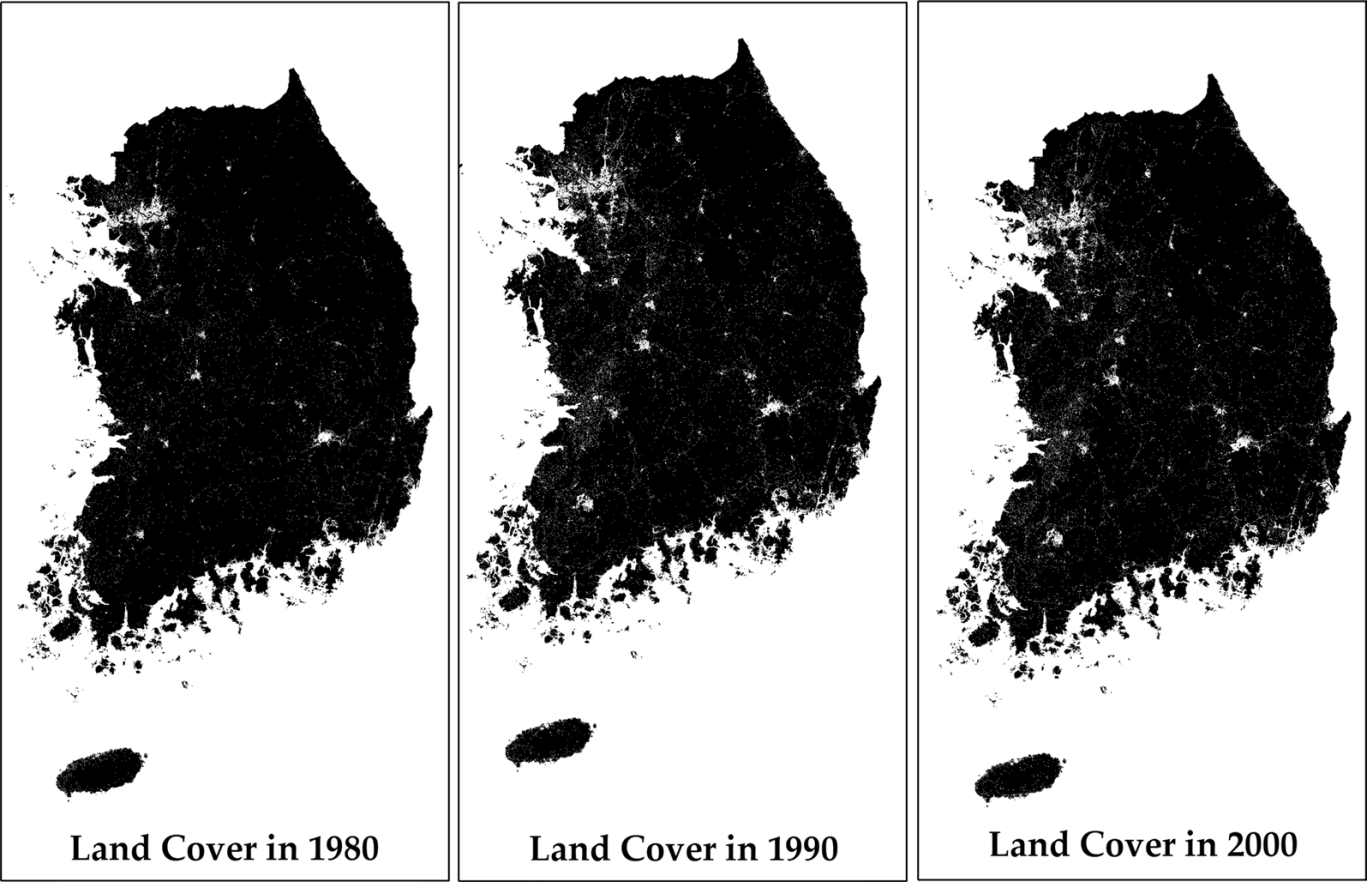
Impervious cover changes in 1980 through 2000

Table 3 explains the changes in area of each land cover since 1980 through 2000. Between 1980 and 1990, developed areas increased by almost 1320 km^2^, and the area with loss of crop was about 2030 km^2^. Another major change appeared in the wetlands. The change in area of wetlands between 1980 and 1990 was approximately 347 km^2^. Increases can be observed in pasture, herbaceous, and open water. More interesting results can be found in the comparison between 1990 and 2000. As can be seen, the developed areas continuously show increase and the difference between 1990 and 2000 was about 674 km^2^. Another major change can be found in the forests. Compared to 1990, the year 2000 experienced a significant expansion in forest area, about 1541 km^2^. This could be perceived as a good sign in terms of preserving the natural environment, but 1541 km^2^ does not provide the overall picture of ecological sustainability. Figure 1 illustrates the impervious cover changes in a map format.

**Table 3:** Land cover change of South Korea in 1980, 1990, and 2000

Land cover	1980	1990		2000		
	km^2^	km^2^	Difference (1990–1980)	km^2^	Difference (2000–1990)	Difference (2000–1980)
Developed areas	2,139.74	3,459.03	1,319.29	4,133.05	674.02	1,993.31
Crop	23,919.12	21,890.15	− 2,028.97	21,386.33	− 503.82	− 2,532.80
Forest	67,178.65	67,122.54	− 56.11	68,669.64	1,541.10	1,484.99
Pasture	3,854.21	4,415.02	560.80	2,905.70	− 1,509.32	− 948.52
Wetlands	785.64	438.62	− 347.02	326.42	− 112.20	− 459.22
Herbaceous	1,300.55	1,689.71	389.16	1,629.85	− 59.86	329.30
Open water	2,027.58	2,190.16	162.59	2,160.30	− 29.86	132.73
Total	101,205.49	101,205.22	− 0.27	101,205.29	0.07	− 0.20

Using the information in Tables 2 and 3, ecological opportunity costs can be estimated between the years 1980 and 2000. To properly identify the changes in the costs, all the unit values in Table 2 were converted to $2015 values using the inflation calculator [48]. As previously mentioned, median ecological values are adopted to avoid any extreme cases that may dominate the estimated results.

As seen in Table 4, the entire ecological stock of South Korea in 1980 was about $5780 million, which is for the 25 million acres in terms of area. In 1990, the entire natural capital had increased to $5822 million and it is about $24.4 million increase compared to 1980. This is an interesting result, as the acreage had actually dropped down to 24.1 million acres and was about 854,455 acre decrease. Although the entire area coverage decreased, the opportunity costs increased by $24.3 million. A few reasons can account for this, but it is mainly because some of the ecological features with relatively higher unit costs, such as pasture, had increased compared to 1980. In 2000, however, both the acreage and cost estimates went down substantially. Compared to 1990, ecological costs has decreased by $293.6 million in 2000 and 166,467 acre decrease in its total amount of ecological features. Unlike the changes found in pervious covers, developed areas gradually increased since 1980. As there is no proper way to measure the economic benefits of the increase in developed areas, it will be difficult to compare the estimates directly. One thing that should be noted, however, is that in 1990, the amount of decrease in pervious covers was greater than the amount of increase in developed land cover. Similar result can be found in 2000, but the degree of impact is not as high as in 1990, but when compared to 1980, the results show significant changes.

**Table 4:** Pervious covers and opportunity costs change since 1980 through 2000

Land cover	1980		1990		2000	
	Acreage	Estimates	Acreage	Estimates	Acreage	Estimates
Crop	5,908,023	$132.4M	5,406,867	$121.1M	5,282,423	$118.3M
Forest	16,593,125	$4,079.3M	16,579,267	$4,075.9M	16,959,918	$4,169.5M
Pasture	951,990	$862.8M	1,090,509	$988.4M	717,707	$650.5M
Wetlands	194,053	$279.1M	108,338	$155.8M	80,626	$115.9M
Herbaceous	321,235	$4.9M	417,358	$6.4M	402,573	$6.2M
Open water	500,811	$439.1M	540,970	$474.3M	533,595	$467.8M
Developed	528,516	–	854,380	–	1,020,863	–
Total	24,997,765	$5,797.5M	24,143,310	$5,821.8M	23,976,843	$5,528.2M

Table 5 explains the detailed difference that occurred in terms of ecological costs and land acreage between 1980 and 2000. Despite the fact that the land acreage had reduced, year 1990 gained about $24.4 million in terms of ecological stocks. Unlike the positive changes, year 2000 lost both acreage and costs. The total amount of pervious cover went down by 166,467 acres and the ecological costs were reduced by $293.6 million. This is a significant change considering the changes observed between 1980 and 1990. Also, this can be the real picture of the consequences induced by massive growth, as Korea started its economic growth boom since the beginning of the 1990s. Similar results can be found in the difference between 2000 and 1980. The loss of acreage appeared to be about 492,400 acres and the ecological stock decreased by $269.3 million. It seems that after 1990, South Korea has changed its environmental appearance substantially when compared to 1980.

**Table 5:** Acreage and costs difference between 1980 and 2000

Land cover	Difference 1990–1980		Difference 2000–1990		Difference 2000–1980	
	Acreage	Estimates	Acreage	Estimate	Acreage	Estimate
Developed	+ 325,864.2	N/A	+ 166,483.6	N/A	+ 492,347.8	N/A
Crop	*− 501,156.2*	*− $11.3M*	*− 124,444.2*	*− $2.8M*	*− 625,600.4*	*− $14.1M*
Forest	*− 13,858.9*	*− $3.4M*	+ 380,651.4	+ $93.6M	+ 366,792.6	+ $90.2M
Pasture	+ 138,518.1	+ $125.5M	*− 372,801.6*	*− $337.9M*	*− 234,283.5*	*− $212.3M*
Wetlands	*− 85,714.7*	*− $123.3M*	*− 27,712.4*	*− $39.8M*	*− 113,427.1*	*− $163.1M*
Herbaceous	+ 96,121.9	+ $1.5M	*− 14,784.3*	*− $0.3M*	+ 81,337.6	+ 1.3M
Open water	+ 162,587.7	+ $35.2M	*− 7,375.7*	*− $6.5M*	+ 32,783.5	+ 28.8M
Total (except developed)	− 325,930.7	+ $24.4M	− 166,466.7	− $293.6M	− 492,397.4	− $269.3M

In sum, the results show that since 1980, South Korea has lost about $269.3 million in ecological opportunity costs and 497,400 acres reduction in terms of pervious coverage within the national boundary. This is a substantial result, as it relates to overall sustainability and effective resource management of the society. If Korean society has gained more economic benefits by sacrificing $269.3 million in ecological stock, this can at least be justified for a better quality of life. Also, if the growth has induced more social as well as financial benefits that are worth more than $269.3 million, then it would have been a discreet decision.

Land cover dataset is provided by the government body every 10 years and, for that reason, tracking down each land cover’s change over time would be a difficult task. However, tracing each land cover’s change over time would provide a substantial amount of information. For example, decreases in pasture are approximately equivalent to the increase in forest between 1980 and 2000. This could be a sign of a change in pasture to forest, as forest is often regarded as a more valuable resource providing more productivity. Articulating such aspect would be an important task for future research and would increase the quality of analysis.

### Growth of South Korea between 1980 and 2000

**Fig. 2:**
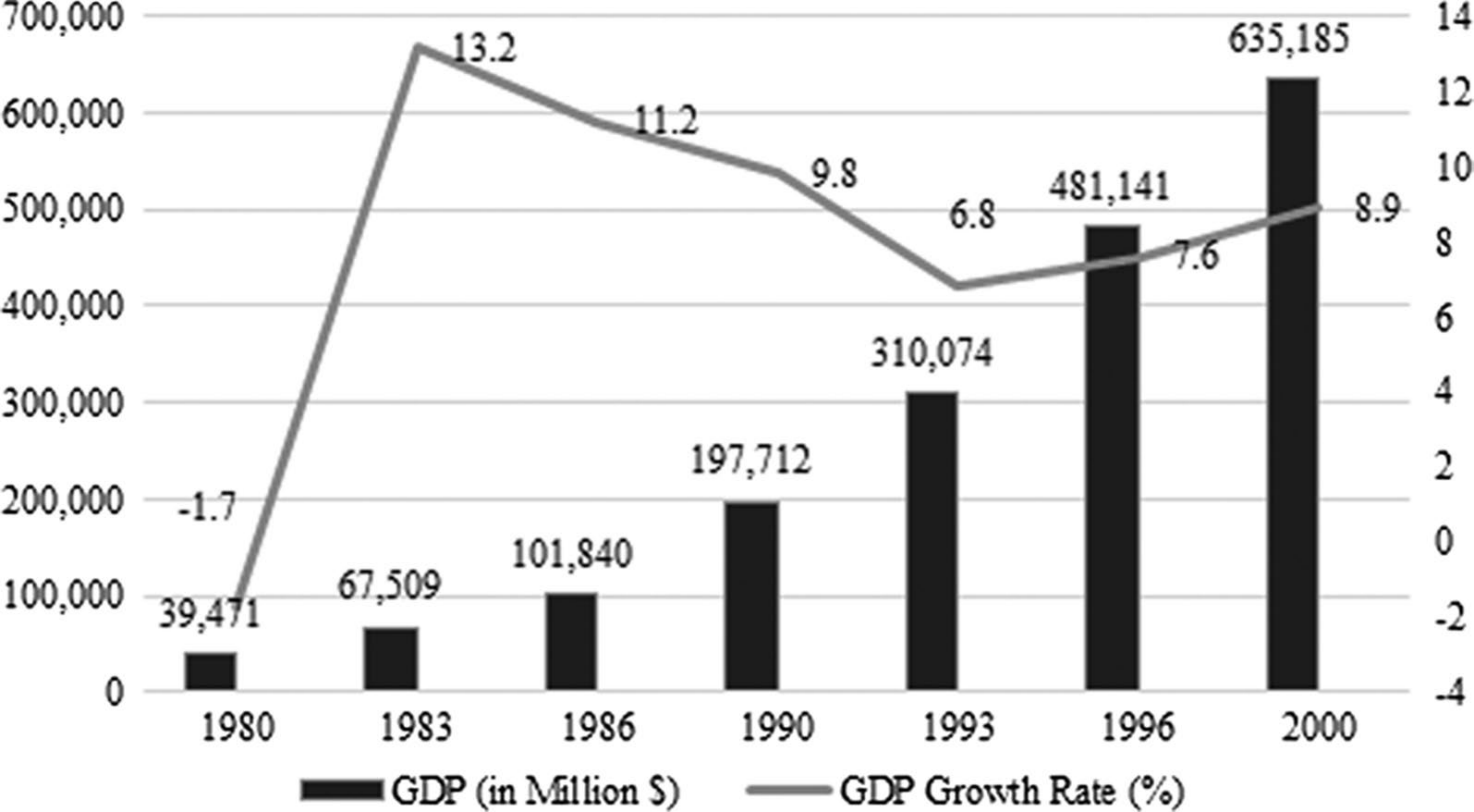
GDP changes of South Korea since 1980

One notable factor to take a look at is the gross domestic products (GDP) changes since 1980. Figure 2 illustrates GDP changes since 1980 through 2000. As can be seen, GDP has continuously increased with substantial amount of gains. Especially during the 1990s, GDP has grown up to almost five times compared to 1980. Accordingly, GDP growth rate is the highest in the 1980s and 1990s. Since 1980, South Korea has gained about $595,714 million in GDP. This is a substantial result, as GDP is highly related to the size of economy and also partially concerns the welfare of citizen’s life. Observation of the records indicates that South Korea has done a successful job over the past decades to expand its size of economy and improve economic stability. At the same time, the entire population has gradually increased. In 1980, the total population of South Korea was about 38,124,000. In 1990, the number went up to 42,869,000, and in 2000 it was 47,008,000. Considering the GDP change that appeared in the same years, population increase is not as dramatic. Looking at the growth rate, it can be understood that the population growth rate has been continuously going down since 1980, reaching under 1% in 2000.

As can be seen in Table 6, by constructing ten major cities with the first- and second-generation city models, South Korea has converted about 380 km^2^ of its natural land cover into impervious surfaces, providing homes to about 929,000 households and 2,390,000 citizens. It is very difficult to directly compare the size of the population with the diminished natural stock during the years, but it is still questionable whether the loss in ecological stock, $269.3 million, can be justified by the new settlements provided to the 2,390,000 residents. Individual ecological cost for 2,390,000 residents is less than $34/person, and this would be even lower considering the fact that the ecological costs is estimated based on the entire land conversion. However, if we understand that our city construction generally lasts for longer than 50 years, the impact of each new resident on the existing natural ecology might be greater than just $134. In addition, the ecological costs were calculated using a conservative measure, median values. If done with the maximum, the estimates may show a slightly different result.

**Table 6:** New city implementation and its population

Cities	Area (km^2^)	Households	Population
1st generation			
Bundang	70	183,000	500,000
Insan	90	220,000	570,000
Pyungchon	51	120,000	350,000
Sanbon	43	57,000	170,000
Jungdong	20	43,000	150,000
2nd generation			
Pangyo	10	30,000	100,000
Dongtan	32	49,000	200,000
Gimpo	4	156,000	150,000
Songdo	51	40,000	100,000
Gwanggyo	12	31,000	100,000
Total	380	929,000	2,390,000

Table 7 shows the difference in the natural capital of South Korea using the maximum ecological values. Unlike the previous results, the ecological stock diminished by about 7% between 1980 and 2000. Using the maximum values changed the results of the declined percentage and the total value of ecological stock. Using the maximum values, the entire stock in 1980 was about $266,841.7 million and $253,423.9 million in 1990. In 2000, the total value went down to $248,397.2 million. This is a substantial difference compared to the results using the median values in Table 5.

**Table 7:** Acreage and costs difference using the maximum values

Land cover	Difference 1990–1980	Difference 2000–1990	Difference 2000–1980
	Estimates	Estimate	Estimate
Developed	N/A	N/A	N/A
Crop	*− $3,311.7M*	*− $822.3M*	*− $4,134.1M*
Forest	*− $148.8M*	*$4,087.5M*	$3,938.6M
Pasture	$1,529.9M	*− $4,117.6M*	*− $2,587.6M*
Wetlands	*− $12,397.5M*	*− $4,008.1M*	*− $16,405.6M*
Herbaceous	$34.2M	*− $5.3M*	$28.9M
Open water	$876.2M	*− $160.9M*	$715.3M
Total (except developed)	− $13,417.7M	− $5,026.8M	− $18,444.5M

The difference between 1980 and 2000 in terms of ecological costs with the maximum unit values is about $18.5 billion, and the population gain during the same period is about 8,884,000. It means that adding 8,884,000 to its total population may have caused South Korean society to spend an amount worth $270 million ecological features using the median ecological values. With the maximum estimates, the difference becomes much higher, $18,445 million, indicating that one population is worth $2076 in terms of environmental costs between 1980 and 2000. In addition, the GDP per capita in 2000 was about $13,512 and the maximum ecological value per capita was $5284, making the difference $8228 more on GDP. On the other hand, per capita GDP in 1980 was $1035 and the ecological value per capita was $6999, making the ecological stock higher by $5694. This is a meaningful result because to gain $12,477 in per capita GDP, South Korean society had to see reductions in ecological stock per capita of $1715. It means if ecological opportunity cost is included as a part of economic products, then the GDP per capita in South Korea should be $10,762, not $12,477, making it similar to the year 1994 values. Table 8 summarizes the result.

**Table 8:** GDP and ecological costs comparison

	1980	2000	Differences
Total population	38,124,000	47,008,000	8,884,000
GDP	$39,471 million	$635,185 million	$595,714 million
GDP per capita	$1035	$13,512	$12,477
Ecological values (median)	$5798 million	$5528 million	− $270 million
Ecological values (maximum)	$266,842 million	$248,397 million	− $18,445 million
Ecological values per capita (median)	$152	$118	− $34
Ecological values per capita (maximum)	$6999	$5284	− $1715

It is hard to precisely compare the ecological stock changes with the GDP increase in South Korea with just simple calculations. As GDP per capita is the sum of annual incomes of all working-age citizens or the sum of all the final goods and services produced in the country, simply comparing GDP changes may not capture precise differences in national economy. In addition, value transfer methods contain several internal assumptions that are partly vulnerable when it comes to methodological justifications. Therefore, calculating ecological values may not provide an in-depth insight into environmental planning of South Korea. Nonetheless, in terms of environmental planning, especially in regional planning, there always is a choice that relevant policy makers and stakeholders could ponder.

What if South Korean government decided to construct just the first-generation new towns? What if we could concentrate more on inner city developments, instead of just expanding new human settlements? Since 2010, old cities in South Korea are experiencing a significant decline in their financial, social, cultural, and physical status. Due to the reason that our birth rate is gradually going down, in fact it reached the lowest in 2017, cities are having a difficult time maintaining their infrastructure and financial status. Many cities in inner areas have observed a massive decline in the number of new students in schools, decrease in their tax revenues, increase in infrastructure maintenance, and surplus in housing supply. These are acceptable consequences, considering our dropped rate in new birth. But if the main purpose of urban planning is to provide a considerate perspective on such sensitive issues, we should at least reconsider our past national development strategies and learn from its lessons to avoid any same mistakes that may happen again. This article is designed to provide such perspectives and to help construct a foundation for a more in-depth analysis on environmental policy between the 1980s and the 2000s in South Korea.

## Conclusions

This study is designed to assess whether South Korean society is moving to a sustainable stage of its development by looking at economic values of natural capital. Since 1980, the society has gained a significant amount of growth in its national economics. In addition, although the growth rate is gradually decreasing, population also added a large portion to its total. Specifically, GDP has increased from about $40 billion to $640 billion and this is a notable achievement because South Korea experienced a tragic war in 1950 and all of these increases were gained in about 50 years.

However, due to its rapid growth, the loss in ecological stock can be observed as well. While the society has gained over 1000% increase in GDP and revenues, the entire natural stock has lost about 5% of its total features using the median values. If calculated with the maximum values, it moves up to a decrease of 7%. This may be perceived as a minimal change, since the society has gained economic expansion higher than 1000%. However, if we try to understand that some of the ecological features do not have natural elasticity and, thus, the loss of ecosystem may become a permanent injury to our natural system, this 7% could be a significant reduction. In addition, now that most of the developing and developed countries have entered into the era of steady growth and experiencing a long-term economic stagnation, constant losses in ecological features can become a factor that should be taken into consideration when elaborating on future growth. This will be especially true for some indices that specifically focus on the quantitative side of development of a society.

The lost services of ecosystem features can be defined in two ways: (1) ecosystem with permanent injury, and (2) ecosystem with natural recovery [10, 11, 25]. The former concerns the service lost due to human interference that will never be restored, making the damages permanent. The latter describes those features also damaged by human activities, but which can recover with natural elasticity. In the latter case, a lost ecosystem will fully bounce back to its previous condition at some point in the future. Urban decisions are closer to a permanent injury, as the initial investments are intended to be long lasting. Therefore, 7% decrease can become a game changer to the human habitat as well as to the natural environment.

South Korean society is experiencing the benefits of rapid economic growth since 1980 and, as a result, the entire population has earned numerous comforts and convenience as an urbanized society. However, if we had estimated the ecological consumptions of rapid growth from the beginning and considered $2076/person for environmental opportunity costs, then the development patterns and other associated urban planning agendas would have shifted accordingly to increase the overall sustainability. Like most developing cities in the world, major cities in South Korea and the central government concentrated their main strategy on economic growth. Doing so stimulated national economy and made it possible to level up the quality of life. If this quality of life needs to be sustained for a long term, then we should focus on our usage on ecological features as their characteristics are completely different from man-made resources.

The methods implemented in this study do not prove whether or not the environmental policies of South Korea are doing a good job based on the classical inferential statistics, nor do they provide truly marketable values of ecological resources because there is no specific market to trade ecological resources with the “invisible-hands”. However, depicting the circumstances of historic natural resource consumption is still a meaningful attempt. By doing so, Korean environmental policies could gain an alternative perspective on assessing its success. For that reason, this study will be dedicated to policy review studies as well as ecological economics projects.
